# Liking of Physical Education and Active Recess Among Chilean Schoolchildren: A National Population-Based Study of Participation Gaps and Reasons for Inactivity

**DOI:** 10.3390/children13091279

**Published:** 2026-09-21

**Authors:** Josivaldo de Souza-Lima, Frano Giakoni-Ramírez, Catalina Muñoz-Strale, Javiera Alarcon-Aguilar, Claudio Farías-Valenzuela, Maribel Parra-Saldías, Daniel Duclos-Bastías, Andrés Godoy-Cumillaf, Eugenio Merellano-Navarro, Tomás Reyes-Amigo

**Affiliations:** 1 Facultad de Educación y Humanidades, Escuela de Ciencias del Deporte, Universidad Andres Bello, Las Condes, Santiago 7550000, Chile; frano.giakoni@unab.cl (F.G.-R.); catalina.munoz@unab.cl (C.M.-S.); javiera.alarcon@unab.cl (J.A.-A.); 2 Departamento de Didáctica de la Expresión Musical, Plástica y Corporal, Facultad de Ciencias de la Educación, Universidad de Granada, 18071 Granada, Spain; 3 Escuela de Ciencias de la Actividad Física, el Deporte y la Salud, Universidad de Santiago de Chile (USACH), Santiago 9170124, Chile; claudio.farias.v@usach.cl; 4 Departamento de Educación Física, Deporte y Recreación, Universidad de Atacama, Copiapó 1530000, Chile; maribel.parra@uda.cl; 5 iGEO Research, Escuela de Educación Física, Pontificia Universidad Católica de Valparaíso, Valparaíso 2340021, Chile; daniel.duclos@pucv.cl; 6 METIS Research Group, Facultad de Negocios y Tecnología, Universidad Alfonso X el Sabio (UAX), 28691 Madrid, Spain; 7 Grupo de Investigación en Educación Física, Salud y Calidad de Vida (EFISAL), Facultad de Educación, Universidad Autónoma de Chile, Temuco 4780000, Chile; andres.godoy@uautonoma.cl; 8 Department of Physical Activity Sciences, Faculty of Education Sciences, Universidad Católica del Maule, Talca 3530000, Chile; emerellano@ucm.cl; 9 Physical Activity Sciences Observatory (OCAF), Universidad de Playa Ancha, Valparaíso 2340000, Chile; tomas.reyes@upla.cl

**Keywords:** physical education, recess, liking of physical education, school health, children, adolescents, Chile

## Abstract

**Highlights:**

**What are the main findings?**

- The adjusted prevalence of inactive recess was 18.2% among students who liked physical education and 56.9% among those who disliked it.
- Disliking physical education was associated with a 3.09-fold prevalence of inactive recess, consistently across sex and age groups.

**What are the implications of the main findings?**

- Enjoyment should be treated as a core implementation outcome of quality physical education, not as an optional by-product.
- Schools need enjoyable, inclusive physical education and accessible games, equipment, and spaces to support movement during recess.

**Abstract:**

**Background/Objectives:** Recess and physical education are complementary school-based opportunities for movement, yet national evidence on how students’ liking of physical education relates to active recess is scarce in Latin America. We examined the association between disliking physical education and inactive recess among Chilean students and described reported reasons for inactive recess. **Methods:** This cross-sectional study used the 2024 Chilean National Survey of Physical Activity and Sports Habits. The primary analysis included 3802 school-attending participants aged 5–17 years with valid responses to the liking of physical education and recess activity items. Inactive recess was defined as reporting no running/jumping, sport, or dance during recess. Modified Poisson regression with normalized survey weights and robust variance estimated adjusted prevalence ratios (aPRs), controlling for age, sex assigned at birth, urban/rural area, socioeconomic status, disability, and region. Region-stratified bootstrap inference used 2000 replicates. **Results:** Weighted active-recess prevalence was 75.8%. Adjusted inactive-recess prevalence was 18.2% (95% confidence interval [CI] 16.0–20.4) among students who liked physical education and 56.9% (95% CI 50.3–63.6) among those who disliked it, an absolute difference of 38.7 percentage points (95% CI 31.6–45.8). Disliking physical education was associated with a prevalence of inactive recess approximately three times higher (aPR 3.09, 95% CI 2.61–3.68); the bootstrap CI was 2.61–3.69. Associations did not differ by sex or age group. Among 977 students with inactive recess, the most frequent reasons were lack of interest (40.3%) and absence of games, equipment, or a suitable place (29.1%). **Conclusions:** Disliking physical education was strongly associated with inactive recess, but temporality cannot be established. Enjoyable, inclusive physical education and better recess opportunities merit joint evaluation in school-based physical activity strategies.

## 1. Introduction

Regular physical activity supports cardiometabolic health, physical fitness, bone health, mental health, and cognitive development across childhood and adolescence [1,2,3]. Current guidance recommends that school-aged children and adolescents accumulate at least 60 min/day of moderate-to-vigorous physical activity, yet insufficient activity remains widespread globally [1,2]. Chile reflects this challenge. Its 2022 national report card assigned low grades to overall physical activity and community/environment indicators, while also identifying persistent implementation gaps despite supportive policies [4]. Throughout this article, physical education refers to the structured curricular subject; physical activity to any bodily movement produced by skeletal muscles that requires energy expenditure; sport to organized, rule-governed physical activity; and physical fitness to a set of attributes, such as cardiorespiratory endurance and muscular strength, that people have or achieve, an outcome rather than a behavior [1,2,5].

Schools are particularly important because they reach children across socioeconomic groups and can provide multiple opportunities for movement throughout the day. A whole-of-school approach combines quality physical education, active recess, extracurricular activities, classroom movement, and active transport rather than relying on a single program [6,7]. Recess, also referred to as break time or playtime in other school systems, denotes the regularly scheduled, non-curricular periods within the school day in which students can move, play, and socialize freely. Recess is not merely a pause between lessons: it offers physical, social, emotional, and cognitive benefits and gives students a setting for self-directed movement and peer interaction [8]. However, recess activity varies substantially. Recent objective and review evidence links recess physical activity with age, sex, available time, facilities, movable equipment, and the design of school spaces [9,10]. Interventions using equipment, playground markings, structured activities, or multicomponent approaches can increase activity in some contexts, although effects remain heterogeneous [11].

Enjoyment may connect formal physical education with voluntary movement elsewhere in the school day. Physical activity enjoyment is associated with self-efficacy, perceived competence, autonomy support, and peer acceptance, and it is consistently related to greater activity [12]. Longitudinal evidence also suggests reciprocal relationships between physical education enjoyment and physical fitness during adolescence [13]. Recent evidence among middle-school students further links perceived physical education competence and enjoyment with persistence and physical activity engagement [14]. These findings support a plausible behavioral pathway: positive experiences in physical education may strengthen competence, confidence, and willingness to move during recess, whereas boredom, embarrassment, excessive difficulty, or poor social experiences may generalize to avoidance. Nevertheless, enjoyment and recess activity can also reflect a shared underlying preference for movement, so cross-sectional associations cannot establish directionality.

Latin American evidence is comparatively limited, and nationally representative estimates spanning childhood and adolescence are rare. A recent Chilean quasi-experimental study found that a 12-week active-recess program based on collaborative games improved several indicators of school coexistence, illustrating that recess can support outcomes beyond movement volume [15]. This question has immediate policy relevance because Chilean Law No. 21,778, published in November 2025, requires schools to promote at least 60 min/day of physical activity, explicitly integrating active methods, recess, and school transport; implementation is scheduled from 2027 and 2028 by educational level [16]. National baseline evidence can help ensure that implementation emphasizes quality, inclusion, and participation rather than minutes alone.

Family socialization adds a further layer to this framework. Parental education operates as a pathway partially independent of household income: through modeling, support, and internalized behavioral patterns, more educated and professionally active parents tend to raise more active children, and recent large-scale evidence links parental education and employment with children’s physical activity levels and weight status [17]. Income-based socioeconomic adjustment therefore captures material resources but not necessarily these family influences, a distinction relevant to the interpretation of the present analysis.

The 2024 Chilean National Survey of Physical Activity and Sports Habits (ENAFyD) provide a unique opportunity to examine school movement experiences in a national sample. Previous ENAFyD analyses addressed physical activity in relation to sleep [18] and neighborhood hostility/park proximity [19], but not the relationship between physical education enjoyment and active recess. Related work by our group has also linked physical activity and sport participation with children’s subjective well-being [20] and documented socioeconomic and rural inequalities in Chilean student outcomes [21]. We therefore aimed to: (1) estimate the association between disliking physical education and physical inactivity during recess (hereafter, inactive recess); (2) test whether this association differed by sex or age group; and (3) describe reasons for inactive recess and for disliking physical education. We hypothesized that students who disliked physical education would have a higher prevalence of inactive recess after sociodemographic adjustment.

## 2. Materials and Methods

### 2.1. Study Design and Data Source

We conducted a cross-sectional secondary analysis of ENAFyD 2024, a population-based household survey commissioned by the Chilean Ministry of Sports to characterize physical activity, sport participation, and related social and environmental factors among residents of Chile [22]. This report follows the principles of the Strengthening the Reporting of Observational Studies in Epidemiology statement [23]. The analytic protocol was finalized after auditing variable coding and skip patterns but before fitting association models for the present research question. It was not externally registered.

### 2.2. Participants and Sampling

The target population comprised children and adolescents aged 5–17 years living in private households in urban and rural areas of all 16 Chilean regions; areas officially classified as difficult to access were excluded from the sampling frame. ENAFyD used probabilistic stratified sampling based on region, age group, sex, urban/rural residence, and socioeconomic status. Trained interviewers administered face-to-face computer-assisted interviews during 2024. The completed 5–17-year sample contained 4150 participants.

The present analysis was restricted to students attending school (p6 = 1). Of 4032 school-attending participants, 77 selected “no response” for recess activities and were excluded. A further 153 had a valid recess response but answered “does not know” for liking of physical education; therefore, 3802 participants formed the primary analytic sample. Within the multiple-response checklists described below, each listed option was coded as reported or not reported, so options that a participant did not select were coded as negative for those options; by contrast, the global “no response” category, indicating that a participant did not answer the item at all, was treated as missing and led to the exclusions described above.

### 2.3. Measures

#### 2.3.1. Survey Instrument

ENAFyD 2024 is a national household survey instrument organized in thematic modules covering sociodemographics, physical activity and sport practice across contexts, school movement behaviors for the 5–17-year population, reasons and barriers, and environmental conditions. It was administered face-to-face by trained interviewers under a documented protocol that included a field pretest and the review and validation of all procedures by the survey’s Ethics Committee [22]. The items analyzed in this study are single categorical items and multiple-response behavioral checklists rather than Likert-type scales; internal-consistency coefficients such as Cronbach’s alpha are undefined for single items and inappropriate for formative checklists, in which selecting one option does not imply selecting another. The items therefore rest on the content and face validity of a purpose-built national statistical instrument, and formal psychometric testing of these specific items has not been published. The verbatim wording of every item used in this study, in Spanish and English, with response options and skip patterns, is provided in Table S3.

#### 2.3.2. Liking of Physical Education

The exposure was the survey item “Do you like the physical education classes at your school?” Responses were yes, no, does not know, or no response. The primary contrast compared “no” (dislikes physical education) with “yes” (likes physical education). “Does not know,” no response, and structural omission among participants not attending school were treated as missing for this estimand. Item wording followed the official ENAFyD 2024 questionnaire [22]. The exposure is thus liking of physical education: a single-item global affective evaluation used as a proxy for the multidimensional enjoyment construct discussed in the literature.

Students who disliked physical education could select multiple reasons: classes are boring; classmates make them feel bad; exercises are too difficult; dislike being watched by classmates; poor relationship with the teacher; feel unwell or have a health problem; does not know; or other. These conditionally administered responses were summarized only within their eligible group.

#### 2.3.3. Recess Activity

Students reported activities usually performed during recess and could select multiple responses: sitting; running or jumping alone or with peers; playing a sport; dancing; playing a board game; using a mobile phone; talking with peers or friends; other; or no response, as specified in the official ENAFyD 2024 questionnaire [22]. Active recess was defined a priori as selecting at least one of running/jumping, sport, or dance. Inactive recess was defined as selecting none of these activities. Sitting or talking could coexist with an active behavior and did not override an active classification. Participants selecting “no response” were excluded. This operational definition captures the absence of the explicitly movement-based options listed in the instrument and should not be interpreted as a comprehensive absence of all physical activity during recess; forms of movement not listed as separate categories, such as walking or informal modified games, may not be captured.

Students classified with inactive recess could report multiple reasons for not playing, practicing sport, or moving school prohibition; no one to play/move with; no games, equipment, or suitable place; peer teasing/bullying; body discomfort; clothing discomfort; lack of interest; or does not know. These items were descriptive and were not entered as predictors because their availability was determined by the outcome-related skip pattern.

#### 2.3.4. Covariates and Secondary Outcomes

Pre-specified covariates were age in years, sex assigned at birth (female/male), urban/rural area, socioeconomic status (high/middle/low), any tutor-reported disability, and region. Age was the tutor-reported age in completed years; area of residence followed the official urban/rural classification of the sampled geographic unit used in the stratified sampling frame; region comprised the 16 administrative regions of Chile; and any disability was defined by a tutor report of at least one visual, hearing, physical, cognitive/intellectual, or other disability. Socioeconomic status was the three-level variable constructed by the survey from total monthly household income, reported in predefined tranches ranging from under $163,000 to over $13,191,000 Chilean pesos with cut-off points that varied by geographic sampling zone, adjusted for household size; the exact construction procedure is documented in the ENAFyD 2024 technical report [22]. All covariates were used as delivered in the public dataset, without recategorization.

Exploratory secondary outcomes were nonparticipation in a school physical activity/sport workshop, nonparticipation in an outside-school workshop, and the official ENAFyD general physical inactivity classification (inactive versus active/partially active). The official index was used as supplied because its exact cross-domain aggregation algorithm was not fully documented in the materials available to the analysts.

### 2.4. Survey Weights and Statistical Analysis

Analyses were performed in Python 3.12.13 using pandas 2.2.3, NumPy 2.5.1, SciPy 1.16.3, statsmodels 0.14.5, patsy, pyreadstat 1.3.5, and matplotlib 3.10.8. The supplied regional weight (pond) had a mean of 1 and summed to approximately 4150; it was normalized to mean 1 for modeling. Weighted percentages are accompanied by unweighted denominators and 95% confidence intervals (CIs).

The primary model used modified Poisson regression with a log link and a robust sandwich covariance to estimate prevalence ratios [24]. The adjusted model included age, sex, urban/rural area, socioeconomic status, disability, and region fixed effects. A weighted logistic model generated standardized marginal probabilities and the absolute prevalence difference and served as a sensitivity analysis for the association scale. Effect modification by sex and by age group (5–11 vs. 12–17 years) was assessed in separate models using global Wald tests.

Sensitivity analyses replaced continuous age with age group; redefined active recess a priori using only running/jumping or sport, removing dance, to verify that the primary association did not depend on any single component; excluded students who simultaneously reported sitting and an active recess behavior; and additionally adjusted for school workshop participation. We performed a 2000-replicate nonparametric bootstrap stratified by region (seed 20260727) for the primary exposure estimate. Exploratory secondary outcome models used the same covariates. Tests were two-sided, and statistical significance was defined as *p* < 0.05 (α = 0.05). The database did not include explicit primary sampling-unit identifiers or the full set of design strata; accordingly, the survey weight alone was not described as reproducing the complete complex design. Region fixed effects, robust variance, and the region-stratified bootstrap were used to evaluate precision under the available design information.

### 2.5. Ethical Considerations

The ENAFyD 2024 protocol was approved by the Ethics Committee of the Chilean Ministry of Sports (Subsecretaría del Deporte; ID 799595-2-LQ24; approved in 2024). Written informed consent was obtained from a parent or legal guardian, and assent was obtained from participating children and adolescents. Data were provided without personal identifiers. The present study analyzed existing de-identified survey data.

### 2.6. Use of Generative Artificial Intelligence

OpenAI Codex was used to assist with statistical code development, quality-control checks, literature searching, and language drafting. The authors are responsible for independently verifying all code, results, references, interpretations, and final wording before submission and take full responsibility for the manuscript. The AI assistance was interactive and iterative rather than based on a fixed set of prompts; reproducibility is therefore guaranteed by the complete, seeded analysis code (bootstrap seed 20260727) provided as Code S4, which reproduces every estimate from the public dataset independently of any AI involvement.

## 3. Results

### 3.1. Analytic Sample and Recess Activity

The primary analytic sample included 3802 students (Figure S1). Their weighted distribution was 52.3% aged 12–17 years, 51.2% female, 82.5% urban, and 31.5% from the low socioeconomic group; 12.9% had any reported disability (Table 1). Overall, 75.8% reported an active recess and 24.2% an inactive recess. Students aged 12–17 years represented 63.3% of the inactive-recess group, compared with 48.8% of the active-recess group. Females represented 56.6% of the inactive-recess group. Physical education was disliked by 15.3% of the analytic sample but by 36.7% of the inactive-recess group.

### 3.2. Liking of Physical Education and Inactive Recess

In the unadjusted modified Poisson model, disliking physical education was associated with 3.20 times the prevalence of inactive recess (95% CI 2.70–3.80). After adjustment for age, sex, area, socioeconomic status, disability, and region, the prevalence ratio was 3.09 (95% CI 2.61–3.68; *p* < 0.001) (Table 2). The region-stratified bootstrap produced a nearly identical percentile CI (2.61–3.69; 2000 successful replicates, no failures).

**Figure 1:**
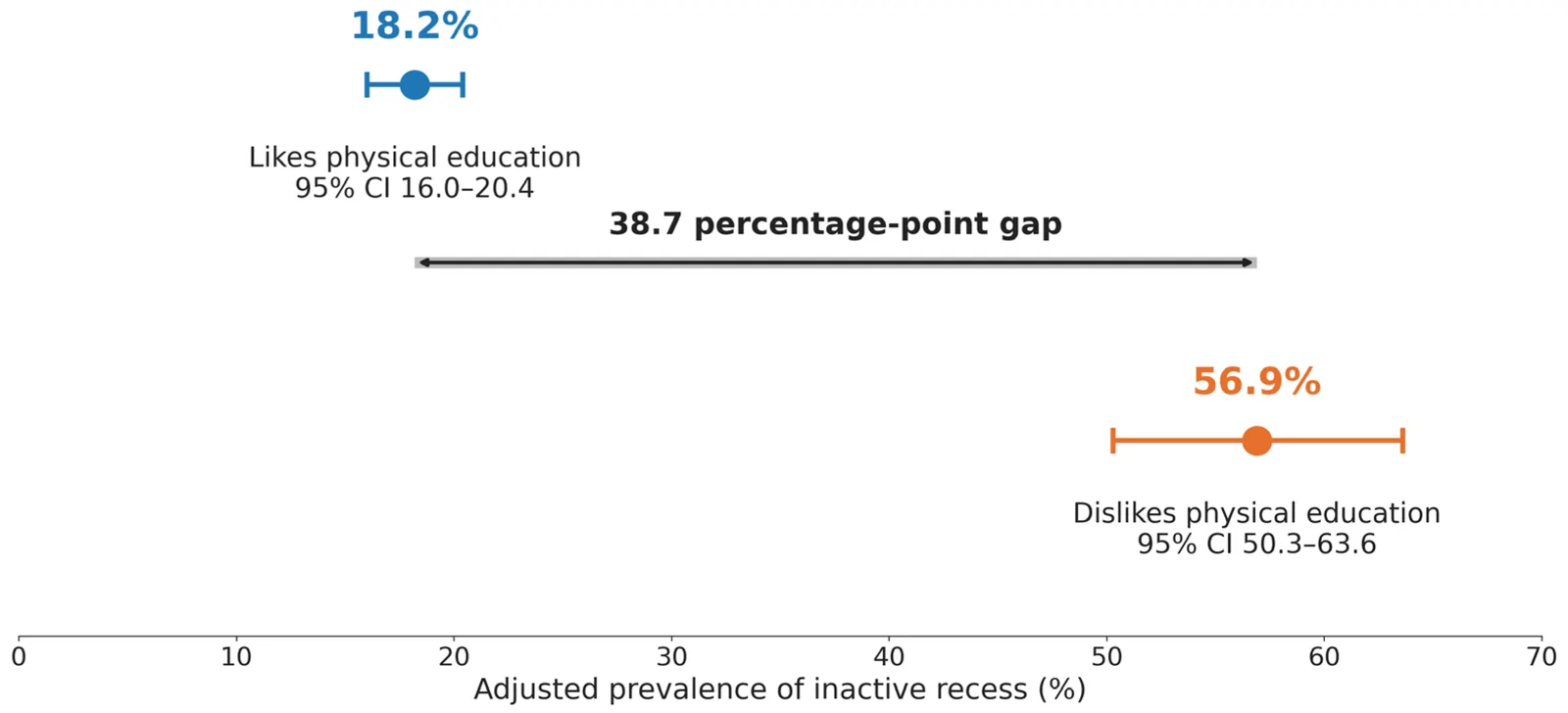
Adjusted prevalence of inactive recess according to liking of physical education. The absolute adjusted difference was 38.7 percentage points. Points are standardized probabilities and error bars are 95% confidence intervals from the weighted logistic model adjusted for age, sex assigned at birth, urban/rural area, socioeconomic status, any disability, and region.

Standardized adjusted inactive-recess prevalence was 18.2% (95% CI 16.0–20.4) among students who liked physical education and 56.9% (95% CI 50.3–63.6) among those who disliked it (Figure 1). The adjusted absolute difference was 38.7 percentage points (95% CI 31.6–45.8).

### 3.3. Effect Modification and Sensitivity Analyses

There was no evidence that the association differed by sex (interaction *p* = 0.844) or age group (interaction *p* = 0.257). The primary estimate remained stable when age was modeled categorically (aPR 3.13, 95% CI 2.64–3.71), dance was excluded from the active-recess definition (aPR 3.14, 95% CI 2.67–3.70), participants reporting both sitting and active behavior were excluded (aPR 3.16, 95% CI 2.70–3.71), and school workshop participation was added to the adjustment set (aPR 3.04, 95% CI 2.54–3.62). The adjusted logistic model yielded an odds ratio of 6.22 (95% CI 4.52–8.57); because inactive recess was common among those disliking physical education, prevalence ratios and standardized probabilities are emphasized.

### 3.4. Reasons for Inactive Recess and Disliking Physical Education

**Figure 2:**
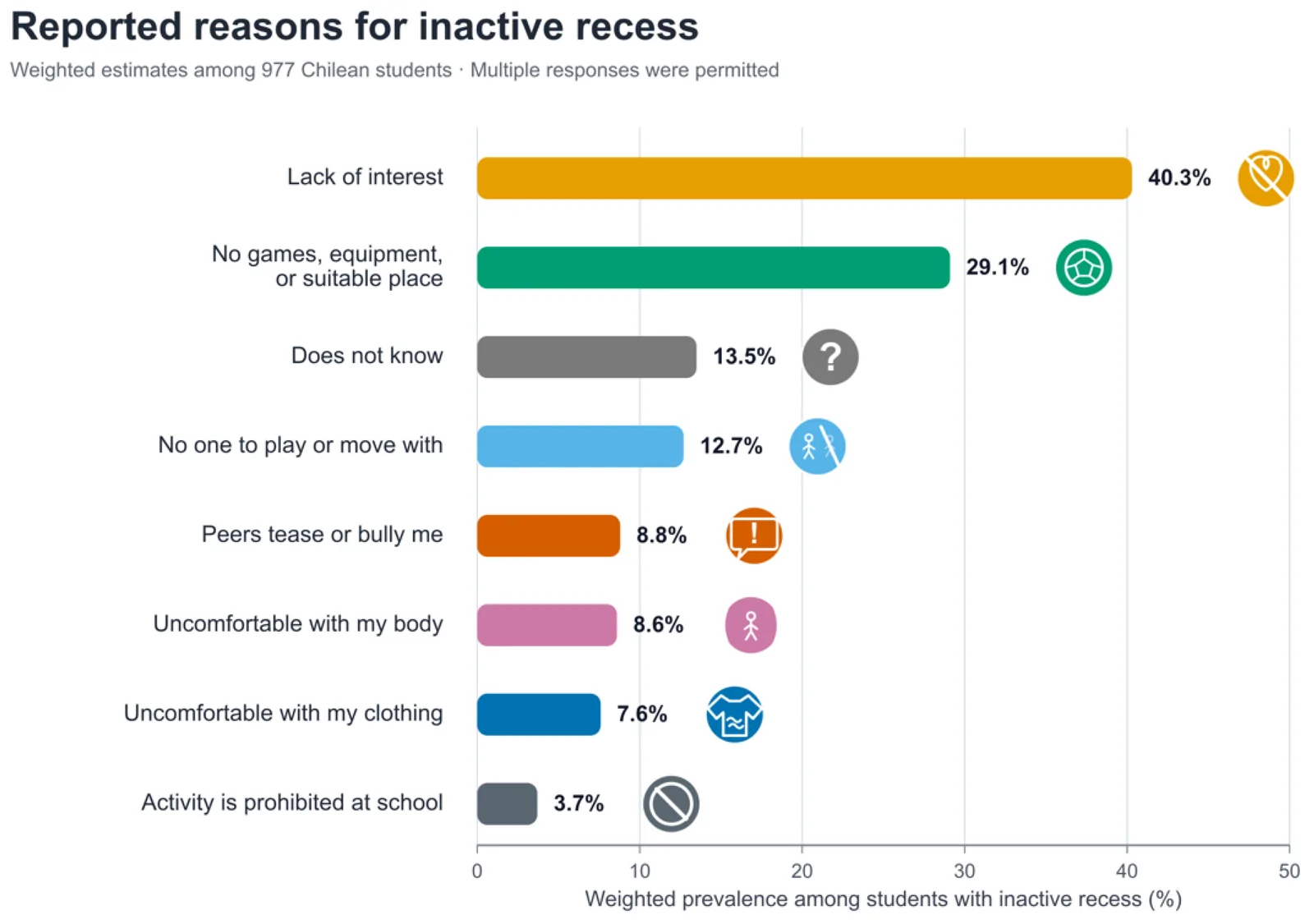
Reported reasons for not playing, practicing sport, or moving during recess among 977 students with inactive recess. Multiple responses were permitted; percentages therefore do not sum to 100.

Among the 977 students with valid inactive-recess responses (the 897 students with inactive recess in the primary analytic sample plus 80 of the 153 students who answered “does not know” to the physical education item; the remaining 73 of those 153 had active recess), the most frequently reported reasons were lack of interest (40.3%, 95% CI 35.2–45.4) and no games, equipment, or suitable place (29.1%, 95% CI 24.3–33.8). Other reasons included not having someone to play or move with (12.7%), peer teasing or bullying (8.8%), body discomfort (8.6%), clothing discomfort (7.6%), and school prohibition (3.7%) (Figure 2).

Among 526 students who disliked physical education, 50.0% described classes as boring. Disliking being watched by classmates was reported by 22.3%, poor relationships with the teacher by 17.9%, exercises being too difficult by 15.9%, and classmates making them feel bad by 12.0%.

### 3.5. Exploratory Secondary Outcomes

Disliking physical education was associated with nonparticipation in an outside-school physical activity or sport workshop (aPR 1.16, 95% CI 1.04–1.30; *p* = 0.008). Associations with nonparticipation in a school workshop (aPR 1.09, 95% CI 0.97–1.23) and general physical inactivity (aPR 1.11, 95% CI 0.97–1.27) were imprecise and compatible with no association. These analyses were exploratory.

## 4. Discussion

### 4.1. Principal Findings

In this national sample of Chilean students aged 5–17 years, disliking physical education was strongly associated with inactive recess. After sociodemographic and regional adjustment, inactive-recess prevalence was approximately three times as high among students who disliked physical education, corresponding to an absolute difference of almost 39 percentage points. The estimate was consistent across robust, bootstrap, and alternative outcome definitions. Although older students and females were more represented among those with inactive recess, the association between liking of physical education and recess activity did not materially differ by sex or age group.

This finding should not be read as evidence that disliking physical education causes inactivity during recess. The exposure and outcome were measured concurrently, and a general preference for activity, perceived competence, prior motor experiences, peer relationships, or other unmeasured factors may shape both. Reverse direction is also plausible: students who habitually avoid active play may find physical education less enjoyable. The result instead identifies a large, policy-relevant clustering of negative experiences across two distinct school-day movement opportunities. The strength of an association between a structured curricular subject and an unstructured, student-directed period is itself informative. One plausible reading is that the pedagogical models in use do not suit a substantial group of students, a reading consistent with the reasons reported below: 50.0% of students who disliked physical education described the classes as boring, 22.3% disliked being watched by classmates, and 15.9% found the exercises too difficult. Our design cannot distinguish this interpretation from shared predisposition or reverse influence, but it identifies teaching quality and pedagogical fit as testable implementation variables.

### 4.2. Comparison with Previous Evidence

The overall active-recess prevalence of 75.8% indicates that most students reported at least one active behavior, but it should not be interpreted as the proportion meeting a duration- or intensity-based recommendation. The updated accelerometer review distinguishes participation in active play from accumulated moderate-to-vigorous physical activity (MVPA) [9]. In a nationally representative Scottish sample, children accumulated a mean of only 3.2 min of MVPA during recess and just 6% met the study’s recess MVPA benchmark; girls accumulated less MVPA than boys [10]. Recent US accelerometry likewise found that recess activity contributed meaningfully to school-day activity and was positively associated with activity during other periods, while emphasizing that cross-sectional data cannot establish compensation or transfer [25]. Self-reported and device-based measures are complementary rather than hierarchical here: accelerometry has known limitations of its own, including wear-time compliance, undercounting of some movement forms, and variation across devices and cut-points, while our measure captures the behavioral reach of active recess in a national sample; the objective estimates therefore provide context rather than a gold standard against which our findings should be downgraded. Its age and sex pattern is compatible with objective evidence, while the absence of interaction in our model means only that the enjoyment association was detectable across the broad sex and age categories examined. This should not be misread as an absence of sex differences in recess behavior: girls showed more inactive recess overall, representing 56.6% of the inactive-recess group versus 49.5% of the active-recess group; what did not differ by sex was the ratio-scale association itself, which roughly tripled the prevalence of inactive recess in both girls and boys.

The magnitude of the association is behaviorally plausible within the recent enjoyment literature, although it is too large to attribute to any single mechanism. A 2024 systematic review identified perceived competence, self-efficacy, autonomy support, social acceptance, and task characteristics as recurrent correlates of physical activity enjoyment [12]. Longitudinal evidence among Finnish adolescents found reciprocal associations between physical education enjoyment and components of physical fitness [13]. Among middle-school students, perceived physical education competence, enjoyment, and persistence were jointly related to physical activity engagement [14]. Together, these studies support a pathway in which repeated experiences of competence and social safety make movement more attractive, whereas difficulty, embarrassment, or exclusion can consolidate avoidance. They also suggest why a uniform activity offer may preferentially engage students who already feel competent.

Recent intervention evidence indicates that enjoyment and motivational climate are modifiable, but behavioral transfer cannot be assumed. A 2024 meta-analysis found that game-based physical education increased enjoyment compared with conventional instruction across children and adolescents [26]. A systematic review and meta-analysis of interventions informed by self-determination and achievement-goal theories reported favorable effects on students’ intentions to remain physically active [27]. In a crossover study published in *Children*, autonomy-supportive teaching improved basic psychological needs, intrinsic motivation, and in-class MVPA [28]. By contrast, a 2023 meta-analysis of out-of-school interventions based on self-determination theory found no pooled improvement in need satisfaction, motivation, or physical activity [29]. Thus, need-supportive and game-based teaching can improve the lesson experience, but evidence is less consistent for activity outside the intervention setting. Our association is compatible with transfer from physical education to recess, but also with shared predisposition, reverse direction, or unmeasured school climate. These candidate mechanisms, competence, autonomy, social safety, and transfer, are hypotheses derived from previous literature: none of these constructs was measured in ENAFyD 2024, and our cross-sectional data are compatible with them without demonstrating them. Transfer, in particular, must be taught rather than assumed: pedagogical models explicitly designed to build transferable skills, autonomy, and game understanding can target the carry-over of movement behaviors to unstructured settings, and implementation should evaluate transfer as an outcome.

The barrier profile further indicates that recess behavior is produced jointly by motivation and opportunity. Lack of interest was the most frequent reason for inactive recess, but nearly three in ten inactive students reported no games, equipment, or suitable place; others identified lack of play partners, bullying, clothing discomfort, or school prohibition. Recent European synthesis describes active recess as dependent on time, space, playground zoning, available activities, supervision, and sensitivity to gendered patterns of use [30]. Current studies of physical education also show that body concerns, peer comparison, and contextual features can influence autonomous motivation and participation [31,32,33,34]. The Chilean questionnaire did not include all potentially relevant conditions, such as weather, device use, changing facilities, or the organization of school spaces. Nevertheless, the co-occurrence of motivational, interpersonal, and environmental responses supports a social-ecological interpretation rather than attributing inactivity solely to individual choice.

Intervention findings also show why equipment should be treated as an enabling condition rather than a complete solution. The 2021 systematic review of elementary-school recess interventions found heterogeneous effects across playground markings, equipment, activity zones, and organized activities [11]. A broader 2024 meta-analysis of controlled school-based interventions similarly reported modest average improvements in physical activity with substantial variation by program content and setting [35]. Reyes-Amigo and colleagues’ 2025 review found generally favorable classroom, executive-function, or fitness outcomes from active breaks, but also considerable heterogeneity in intervention dose and implementation [36]. Most importantly, a recent randomized trial across 21 Dutch primary schools found no improvement in recess physical activity or social-emotional functioning from a standardized one-year playground program, underscoring the importance of context and implementation fidelity [37]. In contrast, the Chilean active-recess program based on collaborative games improved indicators of school coexistence [15]. These mixed findings argue for locally adapted multicomponent strategies rather than assuming that infrastructure or structured games will work uniformly.

Finally, the reasons for disliking physical education add interpersonal and embodied dimensions to the association. Half of the students who disliked physical education described classes as boring, while others reported discomfort being watched, difficult exercises, poor teacher relationships, or classmates making them feel bad. These patterns agree with findings reported by studies in different countries and school systems. In Norwegian adolescents, body-related concerns were associated with lower physical education participation, partly through motivational processes [31]. Among British adolescent girls, peer appearance-related commentary was linked to self-objectification in physical education and indirectly to lower enjoyment [32]. A 2023 systematic review identified appearance, sports competence, teacher behavior, competition, and motivational climate among the correlates of bullying in physical education [33]. Although not directly comparable with our survey-based design, complementary 2025 qualitative evidence suggested that uniform fit, comfort, choice, and self-consciousness could shape girls’ physical education engagement [34]. These studies do not establish the mechanisms operating in ENAFyD, but they make the reported barriers credible and caution against public performance comparisons, inflexible difficulty, compulsory exposure, or clothing rules that intensify social scrutiny.

### 4.3. Implications for School Health and Policy

Chile’s Law No. 21,778 creates a timely opportunity to integrate physical education and recess within a coherent whole-of-school strategy [16]. The law explicitly states that the daily activity requirement complements rather than replaces physical education and includes recess among its implementation settings. This distinction is important: physical education is a curricular learning environment, whereas recess should retain meaningful opportunities for self-selected play, social interaction, and recovery from academic demands. The American Academy of Pediatrics’ updated 2026 policy statement likewise recommends protecting recess and not withholding it for academic or punitive reasons [38]. The WHO school toolkit, recent policy review, CDC recess guidance, and UNESCO’s 2024 global report position quality physical education, recess, classroom movement, active transport, and extracurricular opportunities as complementary components rather than interchangeable minutes [5,6,39,40]. A 2024 review of typical school provision similarly concluded that minor changes to usual physical education, physical activity, and sport provision can be beneficial but that the additive effects of these components remain insufficiently studied [41].

Our findings suggest three priorities for implementation. First, enjoyment and psychological safety should be monitored alongside scheduled minutes and MVPA. Brief anonymous student feedback could identify boredom, excessive difficulty, embarrassment, peer problems, clothing discomfort, and perceived teacher support. Recent reviews show that enjoyment and subjective well-being are relevant implementation outcomes rather than decorative extras [12,42]. Where feasible, schools should combine a brief screening item with an age-appropriate multi-item measure and opportunities for open-ended feedback. Results should be disaggregated by age, sex or gender where available, disability, and socioeconomic context, but not used to grade teachers or students. The purpose is formative: identifying groups for whom increased exposure may reproduce negative experiences instead of building confidence.

Second, physical education should deliberately support competence, autonomy, and relatedness. Practical strategies include progressive task difficulty, meaningful choice, varied individual and cooperative activities, private or self-referenced feedback, and multiple definitions of success. Game-based and motivational interventions provide recent support for these approaches [26,27,28]. The null pooled effect of out-of-school self-determination interventions warrants restraint, however [29]. Implementation should therefore test whether changes in teaching practice improve both enjoyment and voluntary movement elsewhere in the school day rather than treating transfer as automatic. Quality physical education should also deliberately support the transfer of learned skills to recess, home, and community settings.

Third, recess design should expand real opportunities without converting the entire period into compulsory instruction. Accessible portable equipment, usable spaces, active and quiet zones, optional facilitated games, trained supervision, and student co-design can address different combinations of interest, confidence, friendship, and mobility [8,9,10,11,15,30,37,40]. Recess behavior is also shaped by conditions beyond individual preference and school programming, including available space and greenspace, weather and climate, supervision practices, and school policies, so expanding real opportunities means acting on these environmental conditions as well [30,35,39]. The recent Dutch trial shows why standardized programming should be adapted rather than simply replicated [37], while the Chilean collaborative-games study illustrates the potential social value of locally delivered active recess [15]. UNESCO emphasizes adaptable curricula, infrastructure, equipment, trained personnel, inclusion, and meaningful student experiences [5]. The absence of interaction by sex or age in our analysis does not imply identical needs; local consultation is still needed to identify gendered use of space, developmental preferences, clothing concerns, disability access, and culturally appropriate activities.

Pediatricians, school nurses, psychologists, and rehabilitation professionals can support implementation by treating persistent avoidance of school physical activity as a possible signal—not a diagnosis—of motor difficulty, pain, disability, bullying, body-image concern, clothing discomfort, or negative educational experiences [31,32,33,34,38]. Referral pathways should avoid medicalizing ordinary preferences while ensuring that students reporting discomfort, health problems, or peer mistreatment receive appropriate assessment and support. Communication back to schools should focus on reasonable adjustments and participation conditions rather than exemptions alone.

Finally, Chile’s phased implementation offers an opportunity for prospective evaluation. Schools and authorities could monitor not only activity minutes but also reach, enjoyment, perceived safety, exclusion, injuries, fidelity, and differential effects across student groups. Recent intervention syntheses and the new randomized recess trial show that effectiveness depends on context, duration, implementation, and the outcome selected [35,36,37]. Repeated measurements before and after implementation, ideally with school identifiers and objective activity measures in a subsample, would help distinguish whether increased scheduled opportunity translates into equitable participation. Such evaluation would be more informative than assuming that compliance with a 60 min requirement necessarily produces meaningful or enjoyable movement.

### 4.4. Strengths and Limitations

Strengths include a large population-based sample spanning all 16 Chilean regions and the inclusion of both children and adolescents. The analysis explicitly audited questionnaire skips patterns, restricted descriptive reasons to respondents who were eligible to receive each item and did not code structural omissions as negative answers. The revised protocol was finalized before fitting association models for this question. Relative and absolute estimates were presented together, and the primary result was stable across alternative outcome definitions, covariate specifications, a robust variance estimator, and a region-stratified bootstrap. The study also connects two routinely available school experiences and identifies concrete barriers that can inform implementation research. The contribution of this study lies in the formal, national quantification of an association whose direction is intuitive to experienced educators, and in its barrier profile, rather than in the direction of the association itself.

Several limitations qualify the interpretation. First, the cross-sectional design precludes establishing temporality, mediation, or causality. Enjoyment and recess behavior may influence one another, and both may arise from prior motor competence, general activity preference, physical literacy, peer relationships, health, or school climate [12,13,14,27,28,29]. Adjustment reduced measured sociodemographic confounding but cannot address these unmeasured constructs. In particular, the public child-level dataset does not include parental education, so residual confounding by parental education and family socialization, which recent evidence links with children’s activity and weight status [17], is possible; its inclusion in future waves of the survey would strengthen this line of research. Because the exposure and outcome were reported in the same interview, common-method and social-desirability biases may also have inflated or attenuated their association.

Second, liking of physical education and recess activities were measured using brief self-reported items. The binary exposure is not equivalent to a multi-item enjoyment instrument and does not capture ambivalence or day-to-day variation [12,42]. Formal psychometric testing of these specific items, such as test–retest reliability or criterion validity against device-measured activity, has not been published, and the single-item liking measure cannot capture the intensity, stability, or dimensions of enjoyment; nondifferential misclassification from imperfect single items would generally attenuate the association, so the reported estimate is more plausibly an underestimate than an artifact of the instrument. The survey also collected reasons only from students who disliked physical education and only from students with inactive recess, so the positive drivers of enjoyment and of active recess were not recorded, precluding a symmetric analysis. Recess responses identified usual activities but not their frequency, duration, or intensity; accordingly, active-recess prevalence cannot be compared directly with accelerometer-defined MVPA or with compliance with the 60 min/day recommendation [9,10,25]. Recall, comprehension, and the degree of adult assistance may differ between younger children and adolescents. Misclassification is therefore possible, although the consistency of results across alternative active-recess definitions reduces concern that the finding depended solely on dance or on students who also reported sitting.

Third, the supplied analytic file contained no school identifier, school-level policy measures, objective facility audit, primary sampling-unit identifier, or complete set of original design strata. We could not account directly for clustering within schools or distinguish individual experience from institutional context. Survey weights, region fixed effects, robust variance, and a region-stratified bootstrap were used with the information available, but confidence intervals may differ from those produced under the complete original complex design. The weight was treated as an analysis weight after normalization; this does not reconstruct unavailable sampling stages.

Fourth, selection, generalizability, and the fixed-response format require caution. Areas officially classified as difficult to access were outside the sampling frame; 77 school-attending participants lacked a valid recess response, and 153 otherwise eligible participants answered “does not know” for liking of physical education and were excluded, so complete-case estimates may be biased if these students had more negative or irregular school experiences, and transportability to other countries and school systems is not guaranteed. In addition, the reported reasons came from fixed multiple-response lists that identify the prevalence of endorsed barriers but not their severity, ordering, or context, and dimensions such as playground organization, body perception, bullying climate, and uniform comfort were not fully represented in ENAFyD [30,31,33,34]; the barrier findings should therefore guide rather than replace local qualitative work and student participation.

Finally, the binary sex variable available across the full age range cannot characterize gender diversity, and broad interaction tests may have limited power to detect heterogeneous associations within smaller subgroups. Secondary outcomes and sensitivity analyses were supportive but exploratory, and their multiple tests should not be interpreted as confirmatory evidence. Replication with prospective school-linked data, validated enjoyment measures, objective physical activity, and richer measures of teaching and recess context is needed.

## 5. Conclusions

Disliking physical education was associated with a markedly higher prevalence of inactive recess among Chilean students, with consistent estimates across sex, age groups, and sensitivity analyses. Lack of interest and absence of games, equipment, or suitable places were the most frequent reasons for inactivity during recess. These findings do not establish directionality, but they identify positive experiences in physical education and recess opportunities as complementary targets for evaluation. Whole-of-school approaches of this kind gain traction when they are led and embodied by the school’s leadership rather than delegated to individual teachers. As Chile implements a national daily school-activity requirement, success should be judged not only by scheduled minutes but also by whether students experience physical education as enjoyable, inclusive, and transferable to voluntary movement across the school day.

## Figures and Tables

**Table 1:** Weighted characteristics of the primary analytic sample by recess activity.

Characteristic	Overall *n* (%)	Active Recess *n* (%)	Inactive Recess *n* (%)
**Age group**			
5–11	1831 (47.7)	1468 (51.2)	363 (36.7)
12–17	1971 (52.3)	1437 (48.8)	534 (63.3)
**Sex assigned at birth**			
Female	1923 (51.2)	1437 (49.5)	486 (56.6)
Male	1879 (48.8)	1468 (50.5)	411 (43.4)
**Area**			
Urban	3195 (82.5)	2461 (83.1)	734 (80.6)
Rural	607 (17.5)	444 (16.9)	163 (19.4)
**Socioeconomic status**			
High	636 (20.6)	492 (20.5)	144 (20.8)
Middle	1862 (47.9)	1409 (48.5)	453 (46.2)
Low	1304 (31.5)	1004 (31.0)	300 (33.0)
**Any disability**			
No	3374 (87.1)	2581 (86.6)	793 (88.4)
Yes	428 (12.9)	324 (13.4)	104 (11.6)
**Physical education**			
Likes PE	3276 (84.7)	2668 (91.5)	608 (63.3)
Dislikes PE	526 (15.3)	237 (8.5)	289 (36.7)

Values are unweighted *n* and weighted column percentage. The analytic sample included 3802 students: 2905 with active recess and 897 with inactive recess. Percentages may not total 100 because of rounding. Age group is reported in years. Socioeconomic status is the three-level classification (high/middle/low) derived by the survey from total household income and household size.

**Table 2:** Association between liking of physical education and inactive recess.

Model or Contrast	Estimate	95% CI	*p*
Modified Poisson, crude	PR 3.20	2.70–3.80	<0.001
Modified Poisson, adjusted	aPR 3.09	2.61–3.68	<0.001
Region-stratified bootstrap	aPR 3.09	2.61–3.69	<0.001
Adjusted probability: likes PE	18.2%	16.0–20.4	Reference
Adjusted probability: dislikes PE	56.9%	50.3–63.6	<0.001
Adjusted prevalence difference	38.7 pp	31.6–45.8	<0.001

Reference exposure: likes physical education. The adjusted model controlled for age, sex assigned at birth, urban/rural area, socioeconomic status, any disability, and region. Bootstrap: 2000 region-stratified replicates, seed 20260727, no failed fits. aPR, adjusted prevalence ratio; CI, confidence interval; PE, physical education; pp, percentage points; PR, prevalence ratio.

## Data Availability

The data originate from ENAFyD 2024. Owing to confidentiality and data-use conditions established by the Chilean Ministry of Sports, participant-level data are not publicly distributed with this article. Access may be requested through the Ministry’s applicable data-access procedure or from the corresponding author, subject to authorization.

## Supplementary Materials

The following supporting information can be downloaded at https://www.mdpi.com/article/10.3390/children13091279/s1. Figure S1: Flow of the analytic sample; Table S1: Sensitivity analyses and bootstrap results; Table S2: Exploratory secondary outcome models; Table S3: The verbatim survey items in Spanish and English with response options and skip patterns; and Code S4, the complete seeded analysis code (bootstrap seed 20260727), which reproduces every estimate from the public dataset. The de-identified participant-level dataset is not included because access is governed by the Chilean Ministry of Sports.

## Abbreviations

CI, confidence interval; ENAFyD, Encuesta Nacional de Actividad Física y Deporte; MVPA, moderate-to-vigorous physical activity; PA, physical activity; PE, physical education; aPR, adjusted prevalence ratio.
