# Supplementary material for: Liking of Physical Education and Active Recess Among Chilean Schoolchildren: A National Population-Based Study of Participation Gaps and Reasons for Inactivity
Source: Children (Basel). 2026 Sep 21;13(9):1279. doi: 10.3390/children13091279 (PMC13605189; doi:10.3390/children13091279)
Supplement: Supplementary file 1 [file children-13-01279-s001.zip › Code_S4.pdf]

Code S4. Reproducible analysis code

Liking of Physical Education and Active Recess Among Chilean Schoolchildren: A National Population-Based Study of Participation Gaps and Reasons for Inactivity

Python script (bootstrap seed 20260727) that reproduces every estimate reported in the manuscript from the public ENAFyD 2024 child-level dataset. The code is reproduced below as plain text; it can be run by saving its content as a file with a .py extension.

```
# ============================================================================
# Supplementary Material S4. Reproducible analysis code
# Manuscript: Liking of Physical Education and Active Recess Among Chilean
# Schoolchildren (Children, MDPI; manuscript children-4511645)
#
# Data: public child-level dataset (5-17 years) of ENAFyD 2024 (BBDD_NNA.sav,
# n = 4150, 335 variables), Ministry of Sports, Chile.
# Requirements: pandas, numpy, statsmodels, pyreadstat. Seed: 20260727.
#
# Reproduction status (verified on the public dataset, August 2026):
#   Exactly reproduced: participant flow (4150/4032/3955/3802; 977/897);
#   crude PR 3.20 (2.70-3.80); adjusted PR 3.09; bootstrap aPR CI 2.61-3.69
#   (0 failed fits); standardized prevalences 18.2% and 56.9% and their
#   38.7 pp difference (from the weighted logistic model, as stated in
#   Table 2 and Figure 1); all sensitivity analyses (3.14, 3.16, OR 6.22);
#   sex-interaction p = 0.844; all weighted reason percentages.
#   Tolerance notes: the upper analytic CI limit of the adjusted PR rounds
#   to 3.67 here versus 3.68 in the manuscript (boundary rounding, <=0.01);
#   the percentile limits of the bootstrap CI for the prevalence difference
#   vary by <0.5 pp with resampling implementation details.
# ============================================================================

import numpy as np
import pandas as pd
import pyreadstat
import statsmodels.api as sm

RUTA = "/content/drive/MyDrive/ENAFyD_NNA/BBDD_NNA.sav"
SEED = 20260727
N_BOOT = 2000

df, meta = pyreadstat.read_sav(RUTA)
print("Loaded:", df.shape)
s = lambda col: df[col].astype(str).str.strip()

# --- Participant flow (checklists: option k marked = "k.1"; global no-response
# p38_9 = "9.1" treated as missing and excluded) ---
attends = df["p38_9"].notna()
no_resp = s("p38_9").eq("9.1")
valid_recess = attends & ~no_resp
active = s("p38_2").eq("2.1") | s("p38_3").eq("3.1") | s("p38_4").eq("4.1")
inactive = valid_recess & ~active
p35 = s("p35")
likes = p35.isin(["1", "1.0"])
dislikes = p35.isin(["2", "2.0"])
analytic = valid_recess & (likes | dislikes)
print("Attending school:", int(attends.sum()))
print("Excluded (recess no-response):", int((attends & no_resp).sum()))
print("Valid recess:", int(valid_recess.sum()))
print("Inactive recess, all valid (expected 977):", int(inactive.sum()))
print("Analytic sample (expected 3802):", int(analytic.sum()))
print("Inactive in analytic (expected 897):", int((analytic & inactive).sum()))

# --- Analytic dataset (region kept as text labels) ---
d = pd.DataFrame({
    "y": inactive[analytic].astype(int).values,
    "x": dislikes[analytic].astype(int).values,
    "age": pd.to_numeric(df.loc[analytic, "p4"], errors="coerce").values,
    "female": (pd.to_numeric(df.loc[analytic, "p5"], errors="coerce") == 1).astype(int).values,
    "rural": (pd.to_numeric(df.loc[analytic, "p3"], errors="coerce") == 2).astype(int).values,
    "nse": df.loc[analytic, "nse"].astype(str).values,
    "region": df.loc[analytic, "p1"].astype(str).str.strip().values,
    "w": pd.to_numeric(df.loc[analytic, "pond"], errors="coerce").values,
})
any_dis = np.zeros(len(df), dtype=bool)
for c, marked in {"p11_1": "1.1", "p11_2": "2.1", "p11_3": "3.1",
```

```python
                  "p11_4": "4.1", "p11_6": "6.1"}.items():
    any_dis |= s(c).eq(marked).values
d["disab"] = any_dis[analytic.values].astype(int)
d = d.dropna().reset_index(drop=True)
print("Model dataset:", d.shape)

def design(dd):
    X = pd.DataFrame({"const": 1.0, "x": dd["x"], "age": dd["age"],
                      "female": dd["female"], "rural": dd["rural"],
                      "disab": dd["disab"]}, index=dd.index)
    X = pd.concat([X,
        pd.get_dummies(dd["nse"], prefix="nse", drop_first=True, dtype=float),
        pd.get_dummies(dd["region"], prefix="reg", drop_first=True, dtype=float)], axis=1)
    return X

def fit_poisson(dd, robust=True):
    m = sm.GLM(dd["y"], design(dd), family=sm.families.Poisson(), var_weights=dd["w"])
    return m.fit(cov_type="HC1") if robust else m.fit()

def fit_logistic(dd, robust=True):
    m = sm.GLM(dd["y"], design(dd), family=sm.families.Binomial(), var_weights=dd["w"])
    return m.fit(cov_type="HC1") if robust else m.fit()

def std_prev(res, dd):
    X1 = design(dd).copy(); X1["x"] = 1.0
    X0 = design(dd).copy(); X0["x"] = 0.0
    w = dd["w"].values
    return (np.average(res.predict(X0), weights=w) * 100,
            np.average(res.predict(X1), weights=w) * 100)

# --- Crude and adjusted modified Poisson ---
Xc = pd.DataFrame({"const": 1.0, "x": d["x"]})
crude = sm.GLM(d["y"], Xc, family=sm.families.Poisson(), var_weights=d["w"]).fit(cov_type="HC1")
pr = np.exp(crude.params["x"]); ci = np.exp(crude.conf_int().loc["x"])
print(f"Crude PR (expected 3.20, 2.70-3.80): {pr:.2f} ({ci[0]:.2f}-{ci[1]:.2f})")
adj = fit_poisson(d)
apr = np.exp(adj.params["x"]); aci = np.exp(adj.conf_int().loc["x"])
print(f"Adjusted PR (expected 3.09, 2.61-3.68): {apr:.2f} ({aci[0]:.2f}-{aci[1]:.2f}); p =
{adj.pvalues['x']:.2g}")

# --- Standardized prevalences from the weighted logistic model (per Table 2) ---
rlog = fit_logistic(d)
p0, p1 = std_prev(rlog, d)
print(f"Standardized prevalence, likes PE (expected 18.2): {p0:.1f}%")
print(f"Standardized prevalence, dislikes PE (expected 56.9): {p1:.1f}%")
print(f"Adjusted difference (expected 38.7 pp): {p1 - p0:.1f} pp")
print(f"Adjusted logistic OR (expected 6.22): {np.exp(rlog.params['x']):.2f}")

# --- Region-stratified bootstrap, single loop, seed 20260727 ---
rng = np.random.default_rng(SEED)
groups = {r: g.index.to_numpy() for r, g in d.groupby("region")}
b_apr, b_diff, failed = [], [], 0
for b in range(N_BOOT):
    idx = np.concatenate([rng.choice(ix, size=len(ix), replace=True) for ix in groups.values()])
    db = d.loc[idx].reset_index(drop=True)
    try:
        b_apr.append(np.exp(fit_poisson(db, robust=False).params["x"]))
        q0, q1 = std_prev(fit_logistic(db, robust=False), db)
        b_diff.append(q1 - q0)
    except Exception:
        failed += 1
    if (b + 1) % 200 == 0:
        print(f"  bootstrap {b + 1}/{N_BOOT}")
print(f"Bootstrap aPR CI (expected 2.61-3.69): {np.percentile(b_apr, 2.5):.2f}-{np.percentile(b_apr,
97.5):.2f}; failed: {failed}")
print(f"Bootstrap CI for prevalence difference (expected 31.6-45.8; see tolerance note):
{np.percentile(b_diff, 2.5):.1f}-{np.percentile(b_diff, 97.5):.1f}")

# --- Sensitivity analyses ---
active_nd = s("p38_2").eq("2.1") | s("p38_3").eq("3.1")
d8 = d.copy(); d8["y"] = (valid_recess & ~active_nd)[analytic].astype(int).values[:len(d8)]
print(f"Sensitivity, dance excluded (expected 3.14): {np.exp(fit_poisson(d8).params['x']):.2f}")
sit = s("p38_1").eq("1.1")
both = (sit & active)[analytic].values[:len(d)]
print(f"Sensitivity, sitting+active excluded (expected 3.16):
{np.exp(fit_poisson(d.loc[~both]).params['x']):.2f}")
```

```python
Xi = design(d); Xi["x_fem"] = Xi["x"] * Xi["female"]
ri = sm.GLM(d["y"], Xi, family=sm.families.Poisson(), var_weights=d["w"]).fit(cov_type="HC1")
print(f"Sex interaction p (expected 0.844): {ri.pvalues['x_fem']:.3f}")

# --- Weighted reasons ---
w_all = pd.to_numeric(df["pond"], errors="coerce")
print("Reasons for inactive recess (weighted %):")
for c in [f"p39_{k}" for k in range(1, 9)]:
    marked = s(c).str.endswith(".1") & inactive
    pct = 100 * w_all[marked].sum() / w_all[inactive & df[c].notna()].sum()
    print(f"  {c}: {pct:.1f}%  {meta.column_names_to_labels[c][:80]}")
print("Reasons for disliking PE (weighted %):")
mask_d = analytic & dislikes
for c in [f"p36_{k}" for k in range(1, 9)]:
    marked = s(c).str.endswith(".1") & mask_d
    pct = 100 * w_all[marked].sum() / w_all[mask_d & df[c].notna()].sum()
    print(f"  {c}: {pct:.1f}%  {meta.column_names_to_labels[c][:80]}")
print("Done.")
```
