# Supplementary material for: Liking of Physical Education and Active Recess Among Chilean Schoolchildren: A National Population-Based Study of Participation Gaps and Reasons for Inactivity
Source: Children (Basel). 2026 Sep 21;13(9):1279. doi: 10.3390/children13091279 (PMC13605189; doi:10.3390/children13091279)
Supplement: Supplementary file 1 [file children-13-01279-s001.zip › Figure S1, Tables S1-S3.pdf]

Supplementary Materials

Liking of Physical Education and Active Recess Among Chilean Schoolchildren: A National Population-Based Study of Participation Gaps and Reasons for Inactivity

Contents: Figure S1, flow of the analytic sample; Table S1, sensitivity analyses and bootstrap results; Table S2, exploratory secondary outcome models; Table S3, the verbatim survey items in Spanish and English with response options and skip patterns; and Code S4, the complete seeded analysis code (bootstrap seed 20260727), provided as a separate file.

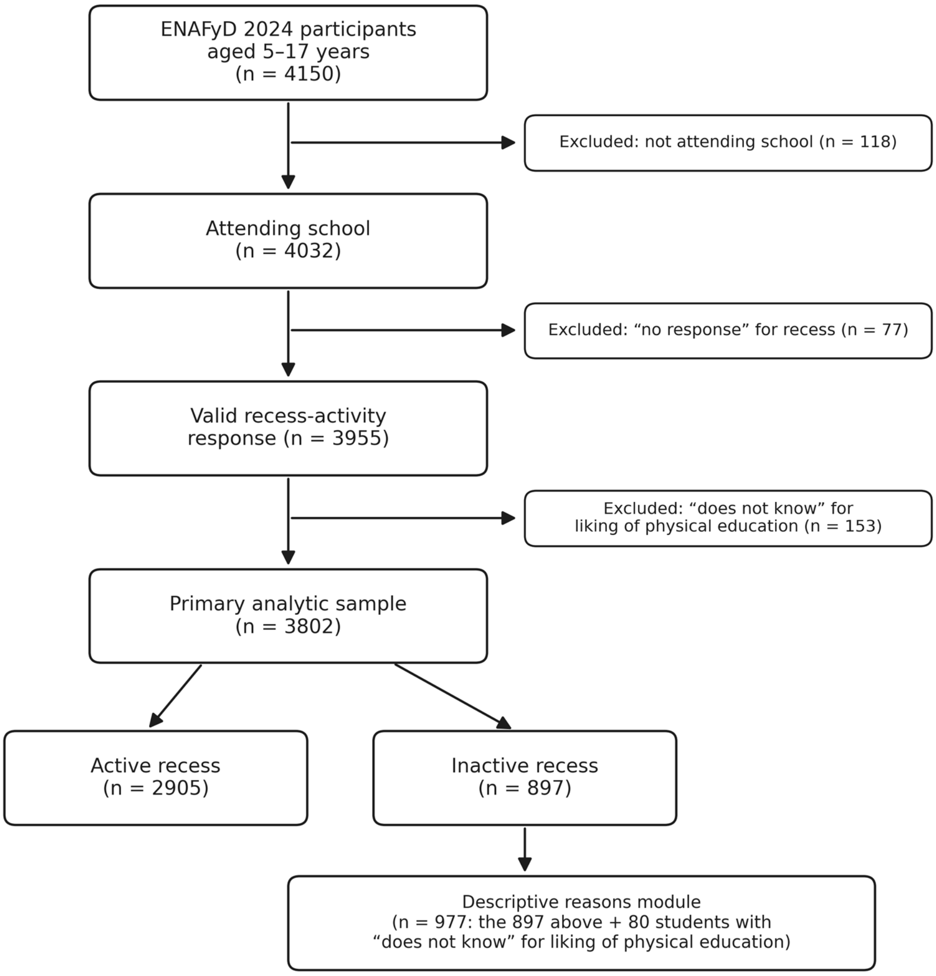

Figure S1. Flow of the analytic sample.

Table S1. Sensitivity analyses and bootstrap results.

Analysis	Estimate	95% CI	p
Primary adjusted model (modified Poisson)	aPR 3.09	2.61–3.68	<0.001
Region-stratified bootstrap (2000 replicates, seed 20260727, no failed fits)	aPR 3.09	2.61–3.69	<0.001
Age modelled categorically (5–11 vs. 12–17 years)	aPR 3.13	2.64–3.71	<0.001
Dance excluded from the active-recess definition	aPR 3.14	2.67–3.70	<0.001
Students reporting both sitting and an active behavior excluded	aPR 3.16	2.70–3.71	<0.001
School workshop participation added to the adjustment set	aPR 3.04	2.54–3.62	<0.001
Weighted logistic model (association scale check)	OR 6.22	4.52–8.57	<0.001
Standardized adjusted prevalence, likes physical education	18.2%	16.0–20.4	Reference
Standardized adjusted prevalence, dislikes physical education	56.9%	50.3–63.6	<0.001
Standardized adjusted prevalence difference	38.7 pp	31.6–45.8	<0.001

All models were estimated with normalized survey weights and a robust sandwich covariance, adjusting for age, sex assigned at birth, urban/rural area, socioeconomic status, any disability, and region, unless otherwise stated. Standardized prevalences and the prevalence difference were obtained from the weighted logistic model. aPR, adjusted prevalence ratio; CI, confidence interval; OR, odds ratio; pp, percentage points.

Table S2. Exploratory secondary outcome models.

Secondary outcome	aPR	95% CI	p
Nonparticipation in an outside-school physical activity or sport workshop	1.16	1.04–1.30	0.008
Nonparticipation in a school physical activity or sport workshop	1.09	0.97–1.23	>0.05
General physical inactivity (official ENAFyD classification)	1.11	0.97–1.27	>0.05

Models used the same covariate set as the primary analysis. These analyses were exploratory and their multiple tests should not be interpreted as confirmatory evidence. aPR, adjusted prevalence ratio; CI, confidence interval.

Table S3. Verbatim survey items in Spanish and English with response options and skip patterns.

All items belong to the school module for the 5-17-year-old population of the ENAFyD 2024 household questionnaire, administered face-to-face by trained interviewers. In the multiple-response checklists, each listed option is recorded as reported or not reported; the “No responde” (no response) option indicates that the participant did not answer the item and was treated as missing. Original orthography is preserved, including the instrument’s spelling “bullyng” [sic].

Panel A. Exposure: liking of physical education classes

Item p35 (single response; administered to school-attending participants aged 5-17). Spanish: “¿Te gustan las clases de Educación Física de tu colegio?” English: “Do you like the physical education classes at your school?” Response options: Sí (Yes) / No (No) / No sabe (Does not know) / No responde (No response).

Panel B. Outcome: recess activities

Item p38 (multiple response; administered to school-attending participants aged 5-17). Spanish: “¿Qué actividades realizas normalmente en los recreos?” English: “Which activities do you usually do during recess?” Response options (Spanish / English): 1. Estoy sentado/a / I am sitting 2. Corro o salto solo o junto a mis compañeros/as / I run or jump alone or with my classmates 3. Practico algún deporte / I play a sport 4. Bailo / I dance 5. Practico algún juego de mesa / I play a board game 6. Uso el celular / I use my mobile phone 7. Converso con compañeros o amigos / I talk with classmates or friends 8. Otra / Other 9. No responde / No response Active recess was defined a priori as reporting at least one of options 2, 3, or 4; inactive recess as reporting none of them. Participants selecting option 9 were excluded.

Panel C. Reasons for inactive recess

Item p39 (multiple response; administered by skip pattern only to students classified with inactive recess). Spanish: “¿Por qué no realizas actividades como jugar, hacer deporte o moverte durante los recreos?” English: “Why do you not do activities such as playing, doing sports, or moving during recess?” Response options (Spanish / English): 1. Porque está prohibido en el colegio / Because it is forbidden at school 2. Porque no tengo con quién jugar o moverme / Because I have no one to play or move with 3. Porque no hay juego, implementos o un lugar para hacerlo / Because there are no games, equipment, or a place to do it 4. Porque mis compañeros me molestan o hacen bullyng [sic] / Because my classmates tease or bully me 5. Porque me siento incómodo/a con mi cuerpo / Because I feel uncomfortable with my body 6. Porque me siento incómodo/a con mi vestimenta / Because I feel uncomfortable with my clothing 7. Porque no me gusta / Because I do not like it 8. No sabe / Does not know

Panel D. Reasons for disliking physical education

Item p36 (multiple response; administered by skip pattern only to students who answered “No” to item p35). Spanish: “¿Por qué no te gustan las clases de educación física?” English: “Why do you not like physical education classes?” Response options (Spanish / English): 1. Porque son aburridas / Because they are boring 2. Porque mis compañeros me hacen sentir mal / Because my classmates make me feel bad 3. Porque los ejercicios son muy difíciles de hacer / Because the exercises are very difficult to do 4. Porque no me gusta que mis compañeros me observen / Because I do not like my classmates watching me 5. Porque no tengo una buena relación con el profesor/a / Because I do not have a good relationship with the teacher 6. Porque me siento mal o tengo un problema de salud / Because I feel unwell or have a health problem 7. No sabe / Does not know 8. Otro / Other

Panel E. Covariates

Item p4 (Spanish: “¿Cuál es su edad?” / English: “What is your age?”): age in completed years, reported by the tutor. Item p5 (Spanish: “¿Cuál fue su sexo asignado al nacer?” / English: “What was your sex assigned at birth?”): Mujer (Female) / Hombre (Male). Item p3 (Spanish: “¿En qué tipo de área vive dentro de la comuna?” / English: “In what type of area do you live within the municipality?”): Urbana (Urban) / Rural (Rural). Item p1 (Spanish: “¿En qué región vive?” / English: “In which region do you live?”): the 16 administrative regions of Chile. Item p11 (Spanish: “¿El niño, niña o adolescente posee alguna de las siguientes discapacidades?” /

English: “Does the child or adolescent have any of the following disabilities?”; multiple response): visual / auditiva (hearing) / física (physical) / cognitiva-intelectual (cognitive-intellectual) / no, ninguna (none) / otra (other). Any disability was defined as reporting at least one of the listed types. Socioeconomic status (variable nse: Alto/Medio/Bajo, i.e., High/Middle/Low) was constructed by the survey from item p10, total monthly household income in predefined tranches, and item p9, household size (Spanish p10: “Por favor, piense en el ingreso total de su hogar en un mes promedio… ¿En cuál de estos tramos está el ingreso total mensual?” / English: “Please think of your household’s total income in an average month… In which of these tranches is the total monthly income?”). The income tranches presented to respondents ranged from under $163,000 to over $13,191,000 Chilean pesos, with cut-off points that varied by geographic sampling zone as documented in the ENAFyD 2024 technical report. Survey weight: variable pond (ponderador), the expansion weight delivered with the public dataset (mean 1.00 in the child sample).
